# MAIT cell alterations in adults with recent-onset and long-term type 1 diabetes

**DOI:** 10.1007/s00125-021-05527-y

**Published:** 2021-08-04

**Authors:** Isabelle Nel, Lucie Beaudoin, Zouriatou Gouda, Camille Rousseau, Pauline Soulard, Matthieu Rouland, Léo Bertrand, Christian Boitard, Etienne Larger, Agnès Lehuen

**Affiliations:** 1 Institut Cochin, Inserm, CNRS, Laboratory of Excellence Inflamex, Université de Paris, Paris, France; 2 Diabetology Department, Cochin Hospital, AP-HP Centre - Université de Paris, Paris, France

**Keywords:** Autoimmunity, Cytokine, Granzyme, Human, Innate immunity, MAIT cells, Type 1 diabetes

## Abstract

**Aims/hypothesis:**

Mucosal-associated invariant T (MAIT) cells are innate-like T lymphocytes expressing an αβ T cell antigen receptor that recognises the MHC-related 1 molecule. MAIT cells are altered in children at risk for and with type 1 diabetes, and mouse model studies have shown MAIT cell involvement in type 1 diabetes development. Since several studies support heterogeneity in type 1 diabetes physiopathology according to the age of individuals, we investigated whether MAIT cells were altered in adults with type 1 diabetes.

**Methods:**

MAIT cell frequency, phenotype and function were analysed by flow cytometry, using fresh peripheral blood from 21 adults with recent-onset type 1 diabetes (2–14 days after disease onset) and 47 adults with long-term disease (>2 years after diagnosis) compared with 55 healthy blood donors. We also separately analysed 17 women with long-term type 1 diabetes and an associated autoimmune disease, compared with 30 healthy women and 27 women with long-term type 1 diabetes.

**Results:**

MAIT cells from adults with recent-onset type 1 diabetes, compared with healthy adult donors, harboured a strongly activated phenotype indicated by an elevated CD25^+^ MAIT cell frequency. In adults with long-term type 1 diabetes, MAIT cells displayed an activated and exhausted phenotype characterised by high CD25 and programmed cell death 1 (PD1) expression and a decreased production of proinflammatory cytokines, IL-2, IFN-γ and TNF-α. Even though MAIT cells from these patients showed upregulated IL-17 and IL-4 production, the polyfunctionality of MAIT cells was decreased (median 4.8 vs 13.14% of MAIT cells, *p* < 0.001) and the frequency of MAIT cells producing none of the effector molecules analysed increased (median 34.40 vs 19.30% of MAIT cells, *p* < 0.01). Several MAIT cell variables correlated with HbA_1c_ level and more particularly in patients with recent-onset type 1 diabetes. In women with long-term type 1 diabetes, MAIT cell alterations were more pronounced in those with an associated autoimmune disease than in those without another autoimmune disease. In women with long-term type 1 diabetes and an associated autoimmune disease, there was an increase in CD69 expression and a decrease in the survival B-cell lymphoma 2 (BCL-2) (*p* < 0.05) and CD127 (IL-7R) (*p* < 0.01) marker expression compared with women without a concomitant autoimmune disorder. Concerning effector molecules, TNF-α and granzyme B production by MAIT cells was decreased.

**Conclusions/interpretation:**

Alterations in MAIT cell frequency, phenotype and function were more pronounced in adults with long-term type 1 diabetes compared with adults with recent-onset type 1 diabetes. There were several correlations between MAIT cell variables and clinical characteristics. Moreover, the presence of another autoimmune disease in women with long-term type 1 diabetes further exacerbated MAIT cell alterations. Our results suggest that MAIT cell alterations in adults with type 1 diabetes could be associated with two aspects of the disease: impaired glucose homeostasis; and autoimmunity.

**Graphical abstract:**

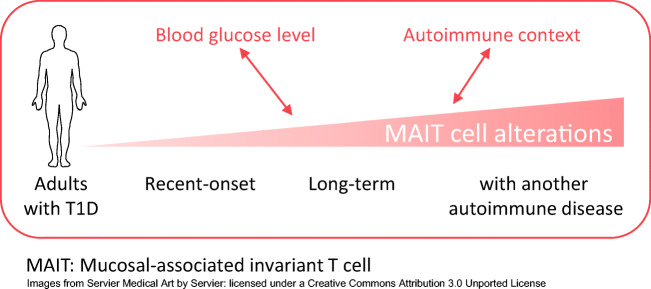

**Supplementary Information:**

The online version of this article contains peer-reviewed but unedited supplementary material. (10.1007/s00125-021-05527-y)

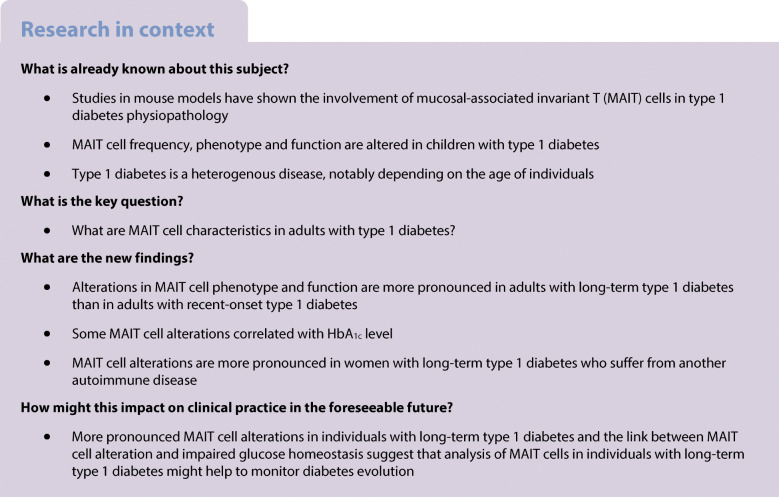



## Introduction

Type 1 diabetes results from the progressive dysfunction and destruction of insulin-secreting pancreatic beta cells [1–3]. It is a multifactorial autoimmune disease in which the innate and the adaptive immune systems are both implicated [3–6]. The resulting hyperglycaemia leads to a requirement for lifelong insulin administration and exposes affected individuals to debilitating micro- and macrovascular complications, which can be fatal [1]. Susceptibility to type 1 diabetes depends on genetic predispositions and environmental factors [1–3]. Half the cases of type 1 diabetes are declared before puberty but the disease can occur at any age [3]. Moreover, approximately 25% of individuals with type 1 diabetes also display a second autoimmune pathology, such as autoimmune thyroiditis or coeliac disease [7, 8].

Mucosal-associated invariant T (MAIT) cells are innate-like T lymphocytes that express a semi-invariant αβ T cell antigen receptor [9–11]. In humans, MAIT cells are found in several tissues and are preferentially located in blood and liver, where they represent 1–10% of T cells [12–14]. They not only recognise a limited number of ligands presented on the MHC-related 1 (MR1) protein, mainly derived from the bacteria riboflavin metabolite pathway, but also can be activated by cytokines [13, 15–19]. Activated MAIT cells produce numerous cytokines (including TNF-α, IFN-γ, IL-4, IL-17 and IL-22) and display cytotoxic activity [12, 13, 20, 21].

Several studies have implicated MAIT cells in autoimmune diseases [22], and we and others recently reported alterations of MAIT cells in children affected by type 1 diabetes [20, 23] and their involvement in mouse models of this pathology [20, 24]. MAIT cell frequency is decreased and they present an activated, exhausted phenotype in the blood of children affected by recent-onset type 1 diabetes [20]. MAIT cells display cytotoxic activity in the pancreas of NOD mice and they can directly kill a human beta pancreatic cell line in vitro [20].

However, type 1 diabetes is an heterogenous disease in individuals, notably depending on age [25–29]. Beta cell destruction occurs faster in children than in adults and ketoacidosis is more frequent at onset in children than in adults [30]. Similarly, MAIT cell frequency and phenotype vary according to age [31, 32]. Such heterogeneity of clinical and biological variables suggests therefore potential differences between children and adults in both involvement of MAIT cells and pathophysiological mechanisms of type 1 diabetes.

Here, we characterised circulating MAIT cells in adults with recent-onset type 1 diabetes and in adults with long-term type 1 diabetes compared with healthy individuals, to determine MAIT cell profiles according to diabetes evolution. We also evaluated the impact of the association of diabetes with another autoimmune disease on MAIT cells in women with long-term type 1 diabetes.

## Methods

### Healthy adult donors and individuals with type 1 diabetes

Peripheral blood samples were collected from 55 healthy blood donors (individuals with diabetes were excluded for blood donation) by the French blood organisation (Etablissement Français du Sang) and from 47 adults with long-term type 1 diabetes (follow-up >2 years), 21 adults with recent-onset type 1 diabetes (Table 1) and 17 adult women suffering from long-term type 1 diabetes and another autoimmune disease (Table 2) from Cochin hospital, Paris. Women in the last group were affected by Graves’ disease (4/17), Hashimoto’s thyroiditis (9/17), autoimmune thyroiditis (3/17) or juvenile polyarthritis (1/17). Type 1 diabetes was diagnosed according to current medical recommendations based on clinical manifestations and biological variables. All individuals with type 1 diabetes presented autoantibodies against at least one of the three antigens GAD, islet antigen 2 (IA2) or zinc transporter 8 (ZnT8) at diagnosis. Individuals with latent autoimmune diabetes in adults (LADA) (age at onset more than 35 years and insulin requirement less than 1 year) or ketosis-prone diabetes were excluded from the study, as well as those with ongoing infectious/inflammatory diseases. Samples were mainly analysed before the COVID-19 outbreak in France (February 2020). Otherwise, blood was taken only from individuals without clinical symptoms of COVID-19 or positive PCR for SARS-CoV-2. Patients with ongoing antibiotic or immunomodulatory treatments were also excluded from this study. Participants with long-term type 1 diabetes were compared with healthy donors of similar age, BMI and sex ratio. The selected participants with long-term type 1 diabetes did not present any renal, cardiac or severe ophthalmological complications.

**Table 1:** Clinical and biological characteristics of individuals with type 1 diabetes and healthy donors

Characteristic	Healthy donors	RO T1D	LT T1D
*n*	55	21	47
Age, years	37.6 ± 13.5 (22.2–67.3)	30.0 ± 12.9 (16.9–69.1)	38.1 ± 14.6 (19.8–74.1)
BMI, kg/m^2^	22.9 ± 3.4 (18.0–32.6)	21.4 ± 2.9 (16.6–27.7)	24.0 ± 4.4 (17.4–39.6)
Sex ratio M/F	25/30	11/10	20/27
T1D duration	–	6 ± 4 days (2–14 days)	18.2 ± 13.2 years (2–53 years)
HbA_1c_, mmol/mol	–	97.1 ± 29.0 (41.0–168.9)	63.0 ± 18.2 (32.2–125.1)
HbA_1c_, %	–	11.0 ± 2.7 (5.9–17.6)	7.9 ± 1.7 (5.1–13.6)
Daily insulin dose, U/kg	–	0.6 ± 0.3 (0.2–1.4)	0.6 ± 0.2 (0.2–1.4)

Data are presented as mean ± SD (range) unless stated otherwise

The adults with recent-onset or long-term type 1 diabetes do not have an associated autoimmune disease

F, female; LT, long-term; M, male; RO, recent-onset; T1D, type 1 diabetes

**Table 2:** Clinical and biological characteristics of healthy women, women with long-term type 1 diabetes or with long-term type 1 diabetes and another autoimmune disease

Characteristic	Healthy donors	LT T1D	LT T1D and another AID
*n*	30	27	17
Age, years	35.8 ± 13.0 (22.2–67.3)	39.4 ± 15.5 (19.8–71.5)	47.8 ± 15.7 (23.5–75.7)
BMI, kg/m^2^	22.3 ± 3.0 (18.0–29.5)	24.5 ± 5.1 (17.4–39.6)	23.7 ± 2.9 (19–28.3)
T1D duration, years	–	18.6 ± 15.3 (2–53)	25.1 ± 14.5 (2–54)
HbA_1c_, mmol/mol	–	65.5 ± 17.9 (39.9–125.1)	64.7 ± 10.7 (48.6–79.2)
HbA_1c_, %	–	8.1 ± 1.6 (5.8–13.6)	8.1 ± 1.0 (6.6–9.4)

Data are presented as mean ± SD (range) unless stated otherwise

AID, autoimmune disease; LT, long-term; T1D, type 1 diabetes

Our study was approved by the Ethics Committee (comité de protection des personnes (CPP) Ile-de-France) (N Eudra CT/ID-RCB: 2014-A00517-40) and all participants provided written informed consent before blood donation.

### Peripheral blood mononuclear cell preparation and flow cytometry analysis

After isolation from fresh blood samples using Ficoll-Paque (Leucosep) tubes, peripheral blood mononuclear cells (PBMCs) were first labelled in PBS containing 5% FCS and 0.1% sodium azide with the following anti-human monoclonal antibodies (mAbs): anti-CD3 (OKT3, cat. no. 317329, RRID:AB_11219196), CD4 (OKT4, cat. no. 317440, RRID:AB_2562912), Vα7.2 (3C10, cat. no. 351715, RRID:AB_2562534), CD161 (HP-3G10, cat. no. 339915, RRID:AB_11142679), CCR6 (G034E3, cat. no. 353410, RRID:AB_10913815), CD56 (HCD56, cat. no. 318340, RRID:AB_2561944), CD69 (FN50, cat. no. 310933, RRID:AB_2561783), CD27 (O323, cat. no. 302835, RRID:AB_2561382) from BioLegend (San Diego, CA, USA); anti-CD8 (SK1, cat. no. 557834, RRID:AB_396892), Programmed cell death 1 (PD1) (MIH4, cat. no. 557860, RRID:AB_2159176), CD25 (M-A251, cat. no. 557741, RRID:AB_396847) from BD Biosciences (San Jose, CA, USA); and anti-CD127 (R34.34, cat. no. A64617, RRID:AB_2833010) from Beckman (Marseille, France).

For B-cell lymphoma 2 (BCL-2) and Ki67 detection after surface staining, PBMCs were resuspended in fixation-permeabilisation buffer (eBiosciences, Thermo Fisher Scientific, Carlsbad, CA, USA) and incubated protected from light at 4°C with mAbs anti-Ki67 (B56, cat. no. 556027, RRID:AB_2266296) from BD Biosciences and anti-BCL-2 (clone 100, cat. no. 658704, RRID:AB_2563152) from BioLegend. PBMCs were then washed with perm wash buffer (eBiosciences).

For detection of cytokines and granzyme B (GzB), PBMCs were activated with phorbol myristate acetate (PMA) (25 ng/ml; Sigma-Aldrich, MO, USA) and ionomycin (1 μg/ml; Sigma-Aldrich) in the presence of brefeldin A (10 μg/ml; Sigma-Aldrich) for 6 h at 37°C in RPMI medium supplemented with 10% FCS. After surface staining, PBMCs were fixed and permeabilised using the Cytofix/Cytoperm kit (BD Biosciences), then washed and stained, protected from light at 4°C, with the following mAbs: anti-IFN-γ (4S B3, cat. no. 506504, RRID:AB_315437), IL-2 (MQ1-17H12, cat. no. 500306, RRID:AB_315093), IL-17 (BL168, cat. no. 512327, RRID:AB_11219603) and TNF-α (Mab11, cat. no. 502937, RRID:AB_2561355) from BioLegend; and anti-IL-4 (8D4-8, cat. no. 560672, RRID:AB_1727547), IL-10 (JES3-19F1, cat. no.554707, RRID:AB_398582) and GzB (GB11, cat. no. 563388, RRID:AB_2738174) from BD Biosciences.

According to the number of PBMCs isolated from each participant or control individual, surface staining was always realised, and when possible intracellular staining of GzB and cytokines after PMA–ionomycin stimulation or of BCL-2 and Ki67 without stimulation. Data acquisition was performed using a BD Biosciences LSR-Fortessa cytometer or a FACSAria III cytometer and results were analysed with FlowJo analysis software V10.1 (Tree Star).

### Statistical and bioinformatic analyses

Multivariable analysis pie charts were built using Excel 2016 (https://www.microsoft.com, Microsoft, Redmond, WA, USA) and FlowJo analysis software V10.1 (https://www.flowjo.com, Becton, Dickinson and Company, San Jose, CA, USA, Tree Star). Principal component analysis (PCA) and correlograms were realised as previously described [33]. Briefly, PCA was processed with FactoMineR package and visualised with Factoextra package. Correlograms were produced with the rcorr function from Hmisc and RcmdrMisc packages to compute pairwise-complete matrices of Spearman’s correlations along with the asymptotic *p* values and visualised with the Corrplot and ggplot2 packages. Datasets were filtered for missing values, and only complete observations were used for Spearman’s correlations. Dplyr library including the tidyverse package was used to sort and filter datasets. Statistical analyses were performed with GraphPad Prism V8.3.0 (https://www.graphpad.com, Graphpad Software, San Diego, CA, USA) or R software V4.0 (https://www.r-project.org/) with RStudio V1.2.5 [34]. Datasets were all tested for normal distribution using the Shapiro–Wilk normality test. All datasets were compared using either non-parametric two-tailed Wilcoxon-Mann–Whitney test or non-parametric Spearman’s correlation test with exact *p* values, as appropriate. Differences with a *p <* 0.05 were considered significant.

## Results

### Circulating MAIT cell phenotype is altered in adults with type 1 diabetes

**Fig. 1:**
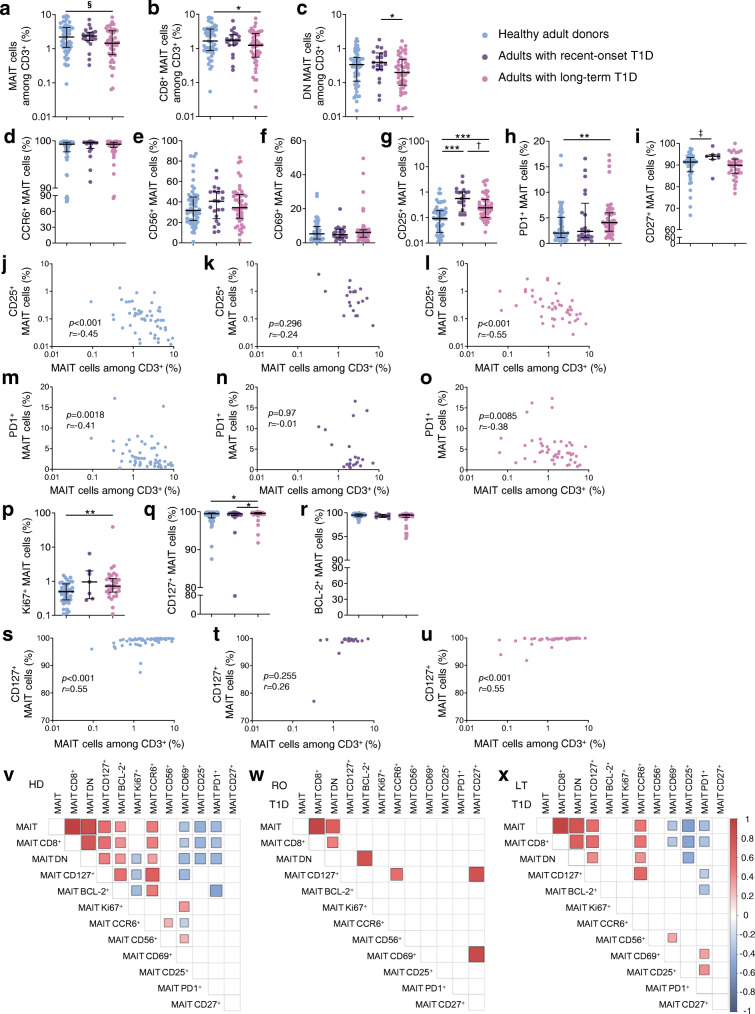
Frequency and phenotype alterations of circulating MAIT cells in adults with type 1 diabetes. PBMCs (5 × 10^6^) were collected from healthy donors (*n* = 55) and from adults with recent-onset (*n* = 21) or long-term (*n* = 47) type 1 diabetes and were analysed by flow cytometry. (**a**–**c**) Circulating MAIT (**a**), MAIT CD8^+^CD4^−^ (**b**) and MAIT CD8^−^CD4^−^ (DN) (**c**) cell frequencies among CD3^+^ cells. (**d**–**i**) Frequencies of CCR6^+^ (**d**), CD56^+^ (**e**), CD69^+^ (**f**), CD25^+^ (**g**), PD1^+^ (**h**) or CD27^+^ (**i**) MAIT cells among total MAIT cells. (**j**–**o**) Correlations between frequency of circulating MAIT cells and frequency of CD25^+^ (**j**–**l**) or PD1^+^ (**m**–**o**) MAIT cells in healthy donors and participants with recent-onset or long-term type 1 diabetes. (**p**–**r**) Frequencies of Ki67^+^ (**p**), CD127^+^ (**q**) or BCL-2^+^ (**r**) MAIT cells among total MAIT cells in healthy donors (*n* = 42, 55, 42 for **p**, **q**, **r**, respectively) and participants with recent-onset (*n* = 7, 21, 7, respectively) or long-term (*n* = 33, 47, 33, respectively) type 1 diabetes. (**s**–**u**) Correlations between frequency of circulating MAIT cells and frequency of CD127^+^ MAIT cells in healthy donors (*n* = 55) (**s**) and participants with recent-onset (*n* = 21) (**t**) or long-term (*n* = 47) (**u**) type 1 diabetes. (**v**–**x**) Correlograms of circulating MAIT cell frequency and marker expression in healthy donors (*n* = 42–55) (**v**) and participants with recent-onset (*n* = 7–21) (**w**) or long-term (*n* = 33–47) (**x**) type 1 diabetes. Only significant Spearman’s correlation coefficients are represented, by colour intensity and square size. Each symbol represents a single individual (**a**–**u**) and small horizontal lines indicate the median with the IQR (**a**–**i**, **p**–**r**). *p < 0.05, **p < 0.01 and ***p < 0.001 (**a**–**i**, **p**–**r**); ^§^*p* = 0.062 (**a**); ^†^*p* = 0.051 (**g**); ^‡^*p* = 0.052 (**i**) (non-parametric two-tailed Mann–Whitney test [**a**–**i**, **p–r**] or Spearman’s correlation test [**j**–**o**, **s**–**x**]). HD, healthy donors; LT, long-term; RO, recent-onset; T1D, type 1 diabetes

We first characterised MAIT cells in fresh peripheral blood samples from adults with long-term type 1 diabetes and from adults with recent-onset type 1 diabetes as compared with healthy adult blood donors. Clinical and biological variables of participants with type 1 diabetes and the healthy donors are reported in Table 1. MAIT cells were identified by flow cytometry as CD161^high^ Vα7.2^+^ T cells (ESM Fig. 1a), identical to Ag-loaded MR1 tetramer identification in blood [33, 35, 36]. In human blood, MAIT cells are divided in two main subsets, CD8^+^ and CD8^−^CD4^−^ (double negative, DN), usually accounting for 80% and 15% of total MAIT cells, respectively (ESM Fig. 1a) [13, 33]. In the participants with long-term type 1 diabetes, CD8^+^ MAIT cell frequency among all T cells was slightly reduced, as compared with healthy adult donors (Fig. 1a, b). There was also a reduction in DN MAIT cell frequency in participants with long-term compared with recent-onset diabetes (Fig. 1c). This suggests altered homeostasis of MAIT cells in individuals with long-term diabetes.

We next analysed the surface phenotype of circulating MAIT cells in the participants with diabetes and the control donors (Fig. 1d–i, ESM Fig. 1b). Frequencies of MAIT cells expressing the tissue-recruitment molecule CCR6 or the adhesion/activation molecule CD56 were similar between the three groups (Fig. 1d, e), suggesting no differences in tissue migration capability. We also quantified frequencies of MAIT cells for expression of markers of early and late activation (CD69 and CD25) as well as markers of sustained activation/exhaustion (PD1). We did not observe any differences in CD69^+^ MAIT cell frequencies between control donors and participants with recent-onset or long-term type 1 diabetes (Fig. 1f). However, the frequency of CD25^+^ MAIT cells was significantly increased in individuals with recent-onset type 1 diabetes and, to a lesser extent, in those with long-term type 1 diabetes (Fig. 1g), when compared with the frequency in healthy donors. Moreover, PD1^+^ MAIT cell frequency was significantly higher in individuals with long-term type 1 diabetes compared with healthy control individuals (Fig. 1h). Expression of the activation marker CD27 on MAIT cells was increased in participants with recent-onset type 1 diabetes compared with healthy donors (*p* = 0.052) (Fig. 1i). Interestingly, both CD25^+^ and PD1^+^ MAIT cell frequencies negatively correlated with MAIT cell frequency in both healthy donors and participants with long-term type 1 diabetes but not in those with recent-onset type 1 diabetes (Fig. 1j–o). This points toward an increased activation status of MAIT cells in individuals with recent-onset type 1 diabetes and also exhaustion of these cells in individuals with long-term type 1 diabetes.

We also analysed expression of markers associated with cell proliferation (Ki67) and survival (CD127 and BCL-2) (Fig. 1p–r and ESM Fig. 1b). Ki67^+^ MAIT cell and CD127^+^ MAIT cell frequencies were significantly higher in individuals with long-term type 1 diabetes compared with healthy control individuals (Fig. 1p, q). No differences in BCL-2^+^ MAIT cell frequency were found between the three groups (Fig. 1r). CD127^+^ MAIT cell frequency positively correlated with blood MAIT cell frequency in participants with long-term type 1 diabetes (Fig. 1u). A similar correlation was observed in healthy control donors but not in participants with recent-onset type 1 diabetes (Fig. 1s, t). Alterations in Ki67 and CD127 expression thus further support specific alteration of MAIT cell homeostasis in individuals with long-term diabetes.

We next performed correlograms with all these MAIT cell variables (Fig. 1v–x). In healthy adult donors, we identified a first cluster of positive correlations between blood MAIT cell frequencies (total MAIT, CD8^+^ MAIT, DN MAIT) and frequencies of MAIT cells expressing tissue residency (CCR6) and survival markers (CD127, BCL-2) (Fig. 1v). Conversely, there were several negative correlations between blood MAIT cell frequencies and frequencies of MAIT cells expressing proliferation (Ki67) and activation markers (CD69, CD25, PD1). In individuals with recent-onset type 1 diabetes, correlations between blood MAIT cell frequencies and frequencies of MAIT cells expressing proliferation, activation and survival markers were lost when compared with healthy control donors (Fig. 1w). However, these clusters were conserved in adults with long-term type 1 diabetes compared with healthy adult donors, with the notable exception of the negative correlations with Ki67^+^ MAIT cell frequency (Fig. 1x). Altogether, our results reveal different MAIT cell alterations in adults with recent-onset or long-term type 1 diabetes.

### Cytokine and GzB production in MAIT cells from adults with type 1 diabetes

**Fig. 2:**
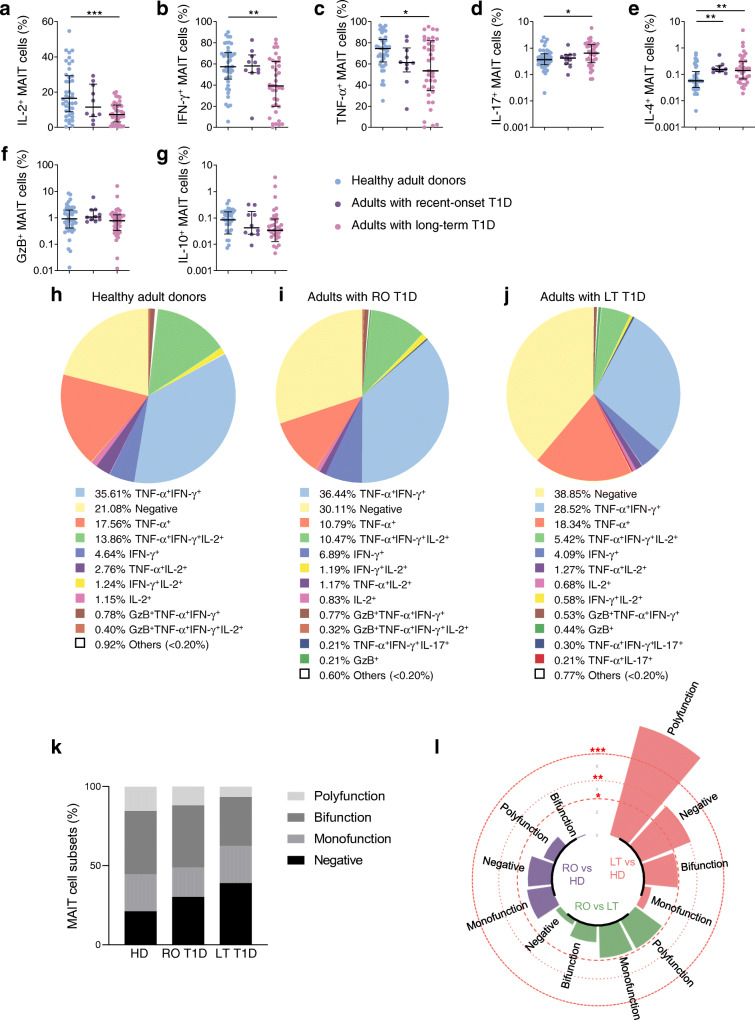
Functional alterations of circulating MAIT cells are correlated in adults with type 1 diabetes. (**a**–**g**) Flow cytometry analysis of IL-2^+^ (**a**), IFN-γ^+^ (**b**), TNF-α^+^ (**c**), IL-17^+^ (**d**), IL-4^+^ (**e**), GzB^+^ (**f**) and IL-10^+^ (**g**) MAIT cells among total MAIT cells in healthy donors (*n* = 42), and adults with recent-onset (*n* = 10) or long-term (*n* = 37) type 1 diabetes. (**h**–**j**) Pie charts showing MAIT cells producing Th1 cytokines, IL-17 and/or GzB, in healthy donors (**h**), adults with recent-onset type 1 diabetes (**i**) or long-term type 1 diabetes (**j**). (**k**) Bar plot representative of cumulative Th1 cytokines, IL-17 and GzB, production by MAIT cells, classified as producing none (negative), one (monofunction), two (bifunction) or more than two (polyfunction) of these factors. (**l**) Circular bar plot represents negative log_*e*_ *p* values of the proportion differences between healthy donors (*n* = 42) and adults with recent-onset (*n* = 10) or long-term (*n* = 37) type 1 diabetes. Red dashed circles represent *p* values. Each symbol represents a single individual and small horizontal lines indicate median with IQR (**a**–**g**). *p < 0.05, **p < 0.01 and ***p < 0.001 (non-parametric two-tailed Mann–Whitney test [**a**–**g**, **l**]). HD, healthy donors; LT, long-term; RO, recent-onset; T1D, type 1 diabetes

We next investigated MAIT cell function by analysing production of Th1 (IL-2, IFN-γ and TNF-α) cytokines, IL-17, GzB, IL-4 and IL-10 after PMA–ionomycin stimulation (Fig. 2a–g). In healthy control individuals MAIT cells produced high levels of Th1 cytokines whereas levels were significantly decreased in individuals with long-term type 1 diabetes (Fig. 2a–c and ESM Fig. 2). IL-17 and IL-4 production by MAIT cells slightly increased in individuals with long-term type 1 diabetes compared with healthy control individuals (Fig. 2d, e and ESM Fig. 2). In participants with recent-onset type 1 diabetes, only IL-4 production was increased. GzB and IL-10 levels in MAIT cells were similar between the three groups (Fig. 2f, g). Of note, cytokine and GzB staining on MAIT cells showed no difference in mean fluorescence intensity (MFI) between the three groups (ESM Fig. 3a).

We further analysed the ability of MAIT cells to produce Th1 cytokines, IL-17 and GzB, by multivariable analysis (Fig. 2h–j). Data showed the functional heterogeneity of MAIT cell subsets in the three groups of participants and, among those producing cytokines, the main subsets produced one, two or three Th1 cytokines. In healthy donors and participants with type 1 diabetes, cytokine-producing MAIT cell subsets were classified as producing none, one (monofunctional), two (bifunctional) or more than two (polyfunctional) different cytokines or GzB to display their functional heterogeneity. Between healthy donors and individuals with type 1 diabetes, there was a progressive increase in the frequency of MAIT cells producing none of the effector molecules analysed (negative subset), from healthy donors to individuals with recent-onset and finally long-term type 1 diabetes (Fig. 2k). Conversely, the frequency of polyfunctional and bifunctional subsets decreased in patients with recent-onset and long-term type 1 diabetes compared with healthy controls (Fig. 2k). This subset distribution differed significantly between healthy donors and individuals with long-term type 1 diabetes (respectively, median 19.30% and 34.40% of MAIT cells for ‘negative’ MAIT cells, *p* < 0.01 and median 13.14% vs 4.8% of MAIT cells for polyfunctional MAIT cells, *p* < 0.001) (Fig. 2l and ESM Fig. 3b).

**Fig. 3:**
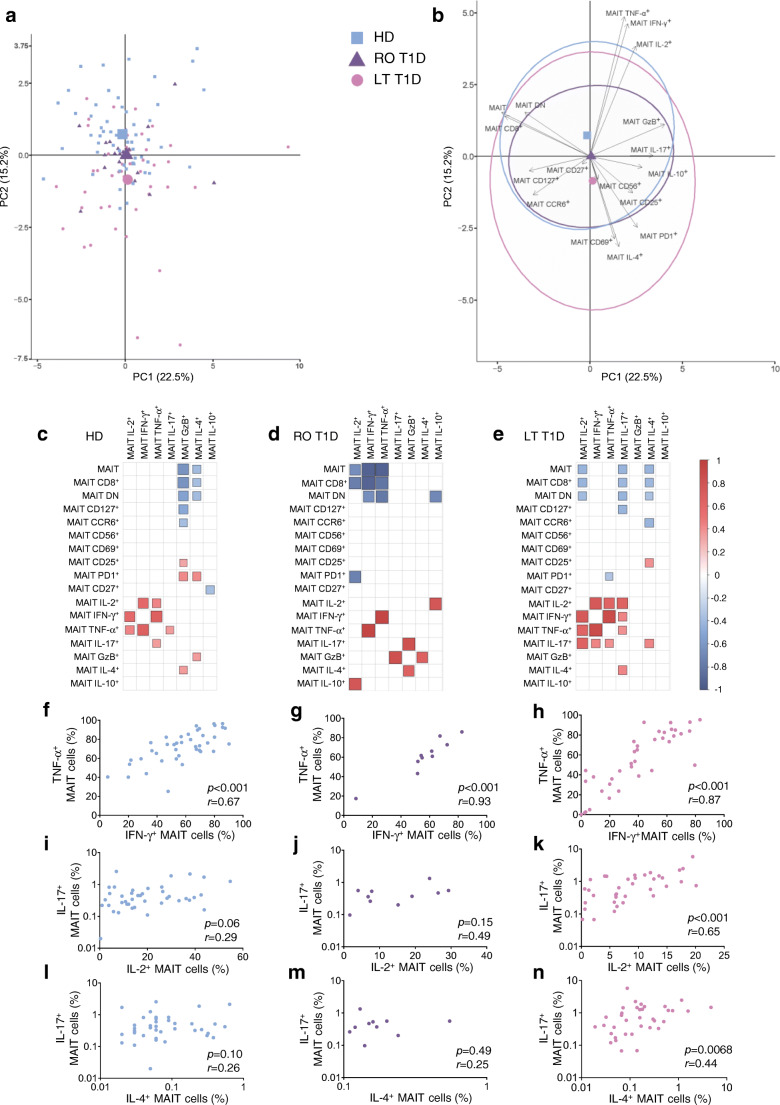
MAIT cell alterations are distinct between adults with recent-onset and long-term type 1 diabetes. (**a**, **b**) PCA of healthy donors (*n* = 42) and adults with recent-onset (*n* = 10) and long-term (*n* = 37) type 1 diabetes, using MAIT cell phenotype and function markers as variables. Each small point represents a single individual and the mean value for each group is represented by a larger symbol (**a**); arrows represent the contribution made by each quantitative variable and concentration ellipses indicate 95% CIs (**b**). (**c**–**e**) Correlograms of circulating MAIT cell phenotype and function in healthy donors (*n* = 33–55) (**c**), adults with recent-onset type 1 diabetes (*n* = 5–21) (**d**) and adults with long-term type 1 diabetes (*n* = 29–47) (**e**). Only significant Spearman’s correlation coefficients are represented by colour intensity and square size. (**f**–**n**) Correlations between TNF-α^+^ and IFN-γ^+^ MAIT cell frequencies (**f**–**h**), between IL-17^+^ and IL-2^+^ MAIT cell frequencies (**i**–**k**), and between IL-17^+^ and IL-4^+^ MAIT cell frequencies (**l**–**n**) in healthy donors (*n* = 42; blue circles), adults with recent-onset type 1 diabetes (*n* = 10; purple circles) or long-term type 1 diabetes (*n* = 37; pink circles). Each point represents a single individual (**f**–**n**). HD, healthy donors; LT, long-term; PC, Principal component; RO, recent-onset; T1D, type 1 diabetes

We next performed a PCA to show the distribution of the three groups of individuals according to MAIT cell frequency, phenotype and functional variables. Participants with long-term type 1 diabetes were more distinctly isolated from healthy control individuals than those with recent-onset type 1 diabetes (Fig. 3a). Decreased Th1 cytokines and increased CD69, PD1 and IL-4 expression were the major contributors to the segregation of adults with long-term type 1 diabetes from heathy donors (Fig. 3b). We next performed correlograms of MAIT cell frequency, phenotype and function in healthy control individuals and participants with type 1 diabetes. A cluster of positive correlations between the three Th1 cytokines was observed in healthy donors and adults with long-term type 1 diabetes, whereas only TNF-α and IFN-γ MAIT cell production correlated with each other in adults with recent-onset type 1 diabetes (Fig. 3c–h and ESM Fig. 3c). Interestingly, MAIT cell IL-17 production positively correlated with MAIT cell TNF-α production in healthy donors and also with other Th1 cytokine production (IFN-γ and IL-2) in individuals with long-term type 1 diabetes. In these individuals, MAIT cell IL-17 production also positively correlated with MAIT cell IL-4 production (Fig. 3c, e, i–n). Correlations between MAIT cell effector molecule production and MAIT cell frequency and phenotype were globally different between the three groups. Altogether, there are distinct MAIT cell phenotypic and functional alterations between individuals with recent-onset and long-term type 1 diabetes.

### MAIT cell alterations correlate with diabetes-associated clinical variables

**Fig. 4:**
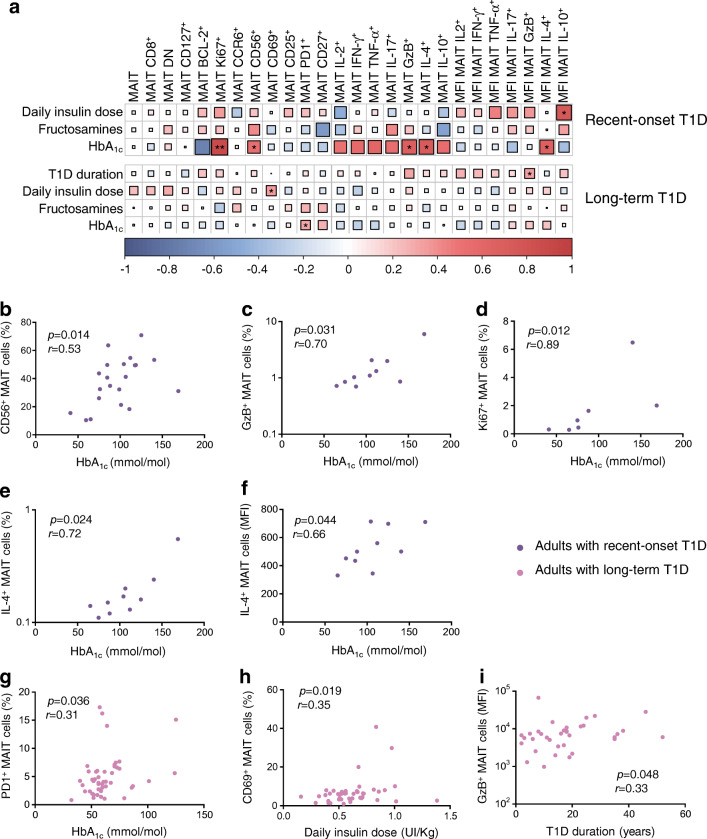
MAIT cell alterations correlated with glycaemic variables in adults with type 1 diabetes. (**a**) Correlograms of circulating MAIT cell frequency, phenotype and function previously analysed and clinical variables (daily insulin dose, fructosamine level, HbA_1c_ level and type 1 diabetes duration) in adults with recent-onset (*n* = 5–21) or long-term (*n* = 24–47) type 1 diabetes. Spearman’s correlation coefficients are represented by colour intensity and square size. Asymptotic *p* values are displayed. All correlations are shown, with asterisks denoting those that are significant. (**b**–**f**) Correlations between CD56^+^ (**b**), GzB^+^ (**c**), Ki67^+^ (**d**) and IL-4^+^ (frequency, **e** and MFI of positive cells, **f**) MAIT cells with HbA_1c_ level in adults with recent-onset type 1 diabetes. (**g**–**i**) Correlations between PD1^+^ MAIT cells and HbA_1c_ level (**g**), between CD69^+^ MAIT cells and daily insulin dose (**h**), and between MFI of GzB^+^ MAIT cells and type 1 diabetes duration (**i**) in adults with long-term type 1 diabetes. Each symbol represents a single individual (**b**–**i**). *p < 0.05 and **p < 0.01 (non-parametric Spearman’s correlation test [**a–i**]). T1D, type 1 diabetes

We next investigated whether MAIT cell frequency, phenotype and functions were associated with clinical variables in adults with type 1 diabetes. In participants with recent-onset type 1 diabetes, correlations were observed between HbA_1c_ level and MAIT cell variables such as CD56^+^, Ki67^+^, GzB^+^ and IL-4^+^ MAIT cell frequencies (Fig. 4a–f). MFI of IL-4 staining also correlated with HbA_1c_ in these individuals. In adults with long-term type 1 diabetes, despite the low variability of HbA_1c_ level, PD1^+^ MAIT cell frequency positively correlated with HbA_1c_ level (Fig. 4a, g). In these individuals, CD69^+^ MAIT cell frequency positively correlated with daily insulin dose (Fig. 4a, g–i). This underlines several associations between MAIT cell alterations and impaired glucose homeostasis in individuals with recent-onset type 1 diabetes and, to a lesser extent, in those with long-term type 1 diabetes.

### Exacerbated MAIT cell alterations in women with long-term type 1 diabetes and another autoimmune disease

**Fig. 5:**
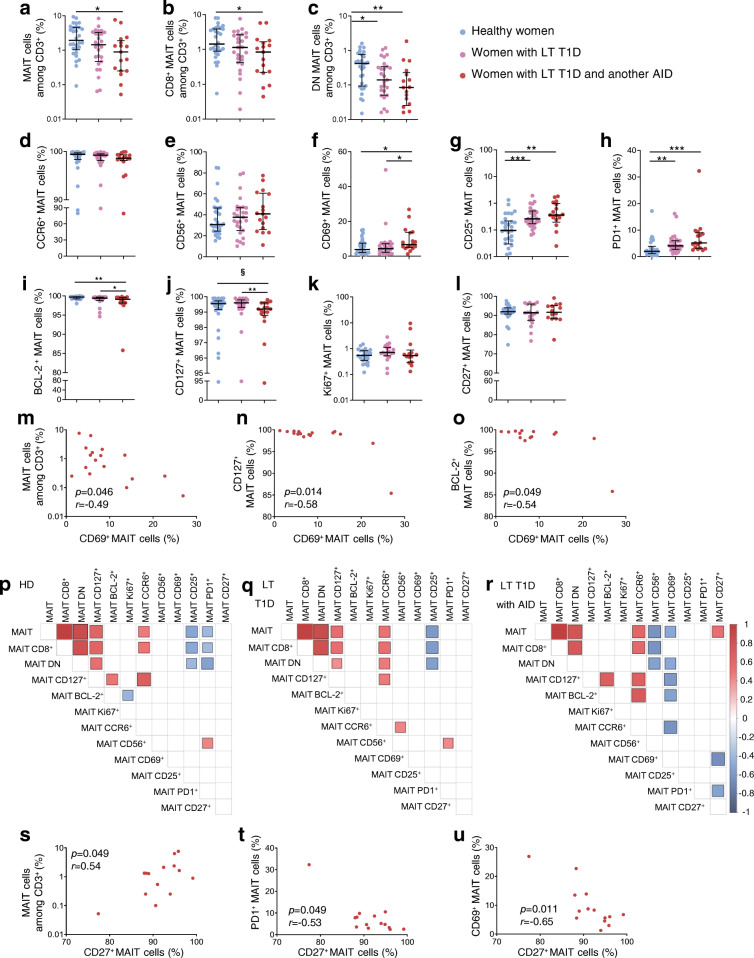
Frequency and phenotype alterations of circulating MAIT cells in women with type 1 diabetes and another autoimmune disease. PBMCs (5 × 10^6^) were collected from healthy female donors (*n* = 30), women with long-term type 1 diabetes (*n* = 27) or women with long-term type 1 diabetes and another autoimmune disease (*n* = 17) and analysed by flow cytometry. (**a**–**c**) Circulating MAIT (**a**), MAIT CD8^+^ CD4^−^ (**b**) and MAIT CD8^−^CD4^−^ (DN) (**c**) cell frequencies among CD3^+^ cells. (**d**–**l**) Frequencies of CCR6^+^ (**d**), CD56^+^ (**e**), CD69^+^ (**f**), CD25^+^ (**g**), PD1^+^ (**h**), BCL-2^+^ (**i**), CD127^+^ (**j**), Ki67^+^ (**k**) and CD27^+^ (**l**) MAIT cells among total MAIT cells. (**m**–**o**) Correlations between circulating MAIT (**m**), CD127^+^ MAIT (**n**) or BCL-2^+^ MAIT (**o**) cell frequencies and CD69^+^ MAIT cell frequency in women with long-term type 1 diabetes and another autoimmune disease (*n* = 17). (**p**–**r**) Correlograms of circulating MAIT cell frequency and phenotype markers in healthy female donors (*n* = 23–30) (**p**), women with long-term type 1 diabetes (*n* = 19–27) (**q**) or long-term type 1 diabetes with another autoimmune disease (*n* = 14–17) (**r**). Only significant Spearman’s correlation coefficients are represented by colour intensity and square size. (**s**–**u**) Correlations between circulating MAIT (**s**), PD1^+^ (**t**) or CD69^+^ (**u**) MAIT cell frequencies and CD27^+^ MAIT cell frequency in women with long-term type 1 diabetes and another autoimmune disease (*n* = 17). Each symbol represents a single individual (**a**–**o**, **s**–**u**) and small horizontal lines indicate the median with the IQR (**a**–**l**). *p < 0.05, **p < 0.01, ***p < 0.001 (**a**–**o**, **s**–**u**) and ^§^*p* = 0.0533 (**j**) (non-parametric two-tailed Mann–Whitney test [**a**–**l**] or Spearman’s correlation test [**m**–**u**]). AID, autoimmune disease; HD, healthy donors; LT, long-term; RO, recent-onset; T1D, type 1 diabetes

We next assessed the impact of another autoimmune disease in association with type 1 diabetes on MAIT cell alterations. Since associated autoimmune diseases mainly occurred in women with diabetes, we compared MAIT cells from women with long-term type 1 diabetes plus another autoimmune disease with those from the other female participants, both healthy and with long-term type 1 diabetes (Table 2). Frequencies of total MAIT cells, CD8^+^ MAIT cells and DN MAIT cells were significantly decreased in women with long-term type 1 diabetes and another autoimmune disease compared with healthy women (Fig. 5a–c). In these women, CD69 expression on MAIT cells was significantly increased compared with healthy women and women with long-term type 1 diabetes (Fig. 5d–h). Expression of survival markers CD127 and BCL-2 by MAIT cells was decreased in women with long-term type 1 diabetes and another autoimmune disease compared with other groups (Fig. 5i–l). Expression of these markers, as well as the frequency of total MAIT cells, negatively correlated with CD69 expression (Fig. 5m–o). Several correlations between CD27 and other MAIT cell variables, such as a positive correlation with MAIT cell frequency and negative correlations with CD69 and PD1 expression by MAIT cells, were only observed in the women with long-term type 1 diabetes and another autoimmune disease (Fig. 5p–u). Altogether, this highlights several modifications of MAIT cells in women with long-term type 1 diabetes and another autoimmune disease that differed from those occurring in either of the other cohorts of women.

**Fig. 6:**
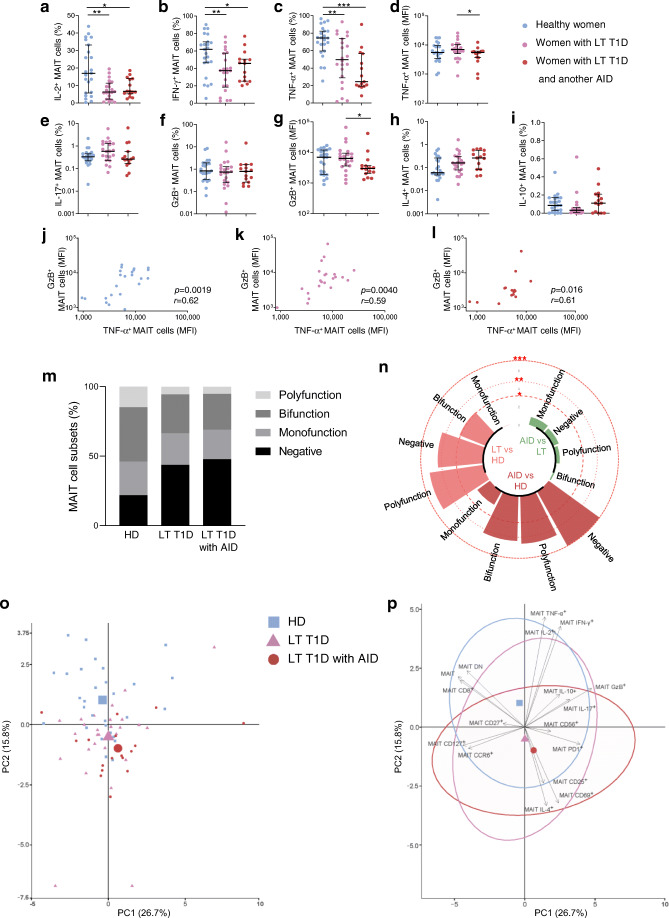
Functional alterations of circulating MAIT cells in women with type 1 diabetes and another autoimmune disease. (**a**–**i**) Flow cytometry analysis of IL-2^+^ (**a**), IFN-γ^+^ (**b**), TNF-α^+^ (**c**), IL-17^+^ (**e**), GzB^+^ (**f**), IL-4^+^ (**h**) or IL-10^+^ (**i**) MAIT cell frequency and MFI of TNF-α^+^ (**d**) or GzB^+^ (**g**). MAIT cell staining in healthy women donors (*n* = 23), women with long-term type 1 diabetes (*n* = 22) and women with long-term type 1 diabetes with another autoimmune disease (*n* = 15). (**j–l**) Correlations between GzB^+^ MAIT cell MFI and TNF-α^+^ MAIT cell MFI in healthy female donors (*n* = 23) (**j**), women with long-term type 1 diabetes (*n* = 22) (**k**) and women with long-term type 1 diabetes and another autoimmune disease (*n* = 15) (**l**). (**m**) Bar plot representative of cumulative production of Th1 cytokines, IL-17 and GzB, by MAIT cells, classified as producing none (negative), one (monofunction), two (bifunction) or more than two (polyfunction) of these factors. (**n**) Circular bar plot represents negative log_*e*_ *p* values of the proportion differences between healthy female donors (*n* = 23), women with long-term type 1 diabetes (*n* = 22) and women with long-term type 1 diabetes and another autoimmune disease (*n* = 15). Red dashed circles represent *p* values. (**o**, **p**) PCA of healthy female donors (*n* = 23), women with long-term type 1 diabetes (*n* = 22) and women with long-term type 1 diabetes and another autoimmune disease (*n* = 15), using MAIT cell phenotype and function markers as variables. Each small point represents a single individual and the mean value for each group is represented by a larger symbol (**o**); arrows represent the contribution made by each quantitative variable and concentration ellipses indicate 95% CIs (**p**). Each symbol represents a single individual (**a**–**l**, **o**) and small horizontal lines indicate the median with the IQR (**a**–**i**). *p < 0.05, **p < 0.01 and ***p < 0.001 (non-parametric two-tailed Mann–Whitney test [**a**–**i**, **n**] or Spearman’s correlation test [**j**–**l**]). AID, autoimmune disease; HD, healthy donors; LT, long-term; PC, Principal component; RO, recent-onset; T1D, type 1 diabetes

Analysis of IL-2, IFN-γ and TNF-α showed that the proportion of MAIT cells producing these cytokines was reduced in women with long-term type 1 diabetes compared with healthy donors regardless of autoimmune disease status (Fig. 6a–c). Among the variables studied, the MFI of MAIT cells after staining was lower for TNF-α and GzB in women with long-term type 1 diabetes and another autoimmune disease compared with women with long-term type 1 diabetes (Fig. 6d, g and ESM Fig. 4a). Of note these two variables correlated with each other in all three groups of women analysed (Fig. 6j–l and ESM Fig. 4b). As previously shown with individuals with long-term type 1 diabetes, women from this group also displayed an elevated frequency of the negative cytokine/GzB-producing MAIT cell subset compared with healthy women (Fig. 6m and ESM Fig. 4c). MAIT cells from women with long-term type 1 diabetes and women with long-term type 1 diabetes and another autoimmune disease presented a similar profile. In both groups, MAIT cells were less multifunctional compared with healthy female donors, with decreased polyfunctional and bifunctional MAIT cell frequencies (Fig. 6n). PCA revealed a better segregation from healthy female donors of women with long-term diabetes and another autoimmune disease than women with long-term diabetes (Fig. 6o, p). MAIT cell (total, CD8^+^, DN) frequencies and CD69 expression contributed to segregation between women with long-term diabetes with or without another autoimmune disease. Therefore, we revealed common and distinct MAIT cell alterations between both groups of women with long-term diabetes.

## Discussion

The present study shows that MAIT cells are altered in adults with type 1 diabetes. These alterations in phenotype and function are more pronounced in adults with long-term type 1 diabetes compared with those with recent-onset type 1 diabetes. Several MAIT cell alterations positively correlated to glucose homeostasis impairment. Moreover, the presence of another autoimmune disease in women with long-term type 1 diabetes exacerbated MAIT cell alterations (ESM Table 1).

In individuals with recent-onset type 1 diabetes, we demonstrated that MAIT cells harboured an activated profile (CD25 and CD27 upregulation) without major alterations in cell homeostasis and function. In contrast, in individuals with long-term type 1 diabetes, as well as CD25, MAIT cells also displayed upregulation of the exhaustion marker PD1 and molecules involved in proliferation (Ki67) and survival (CD127). CD8^+^ and DN MAIT cell frequencies were decreased in these individuals and this increased proliferation and survivability might be a mechanism to compensate for their lower frequencies. MAIT cell frequency negatively correlated with exhausted MAIT cell frequency and positively with CD127^+^ MAIT cell frequency. Such correlations were not observed in individuals with recent-onset type 1 diabetes, suggesting that MAIT cell profiles may differ as the disease evolves. Whether these differences depend on disease history or may predict future complications remains to be investigated.

In our study, we addressed whether these heterogenous MAIT cell alterations were related to diabetes-associated clinical variables. In individuals with recent-onset type 1 diabetes, Ki67^+^, CD56^+^, IL-4^+^ and GzB^+^ MAIT cell frequency positively correlated with blood HbA_1c_ level. Production of IL-4^+^ and GzB^+^ is elevated in MAIT cells from children with recent-onset type 1 diabetes, although GzB^+^ MAIT cell frequency inversely correlates with blood HbA_1c_ [20]. As type 1 diabetes is less aggressive in adults [30], these correlations may reflect a slowly rising blood glucose level before disease diagnosis, linked with progressive alterations of MAIT cells [20]. In contrast, in our study participants with long-term type 1 diabetes, HbA_1c_ levels positively correlated only with PD1^+^ MAIT cell frequency. This could result from a negative impact of the increased blood glucose level on MAIT cell frequency and function as already described in obesity and type 2 diabetes [37, 38]. Alternatively, higher PD1 expression by MAIT cells could reflect a chronic activation status due to increased general inflammation levels in individuals with less-well-controlled diabetes [36–38]. Of note, our present cohort was composed of individuals without complications and who had globally well-controlled blood glucose levels, so it would be of interest to study eventual differences in the status of MAIT cells in uncontrolled type 1 diabetes.

In agreement with this increased PD1 expression, the production of Th1 cytokines was decreased in MAIT cells from individuals with long-term type 1 diabetes. Circulating MAIT cells in humans mainly produce Th1 cytokines, such as IL-2 (18%), IFN-γ (60%) and TNF-α (75%) [13]. Decreased Th1 cytokine production by MAIT cells was associated with increased frequency of MAIT cells that synthesised none of the cytokines analysed nor GzB, and a loss of polyfunctional MAIT cells. Even though there was a global shift of Th1 to IL-17 and IL-4 cytokines by MAIT cells in individuals with long-term type 1 diabetes compared with healthy donors, the production of all these cytokines strongly and positively correlated with each other. These data suggest that among the individuals with long-term type 1 diabetes, some harboured an elevated frequency of MAIT cells producing all cytokines (Th1, IL-17 and IL-4) whereas others possessed MAIT cells with a general low level of cytokine production. Moreover, multivariable analysis showed the preferential production of IL-17 in association with TNF-α and IFN-γ at the cellular level. This is of importance as conventional Th1/Th17 double-producer T cells are highly pathogenic in the context of autoimmune diseases and type 1 diabetes [39–41]. Monitoring MAIT cell cytokine production may therefore help to estimate future complications. Moreover, the phenotype of MAIT cells can be significantly influenced by other immune cell populations such as monocytes and inflammatory cytokines [13, 14, 33, 42]. Similar immune cell crosstalk has been well established in type 1 diabetes [4, 43] and it would be interesting to link these alterations to other differences in immune cells and cytokines between individuals with recent-onset and long-term type 1 diabetes.

Type 1 diabetes is often associated with other autoimmune diseases, particularly in women [44]. The major MAIT cell alterations in women with long-term type 1 diabetes and autoimmune disease concerned MAIT cell homeostasis. Compared with women affected by long-term type 1 diabetes only, MAIT cell frequency and survivability (BCL-2^+^ and CD127^+^ frequency) were significantly decreased. CD69^+^ MAIT cell frequency was increased and negatively correlated with MAIT cell frequency, BCL-2^+^ MAIT cell and CD127^+^ MAIT cell frequencies. Even though CD69 is mainly known as an early activation marker, it is also expressed on T cells under chronic activation [45]. MAIT cells might therefore be under further sustained activation in individuals with diabetes and another autoimmune disease, although confirming this hypothesis would require comparison with individuals affected by an autoimmune disease without type 1 diabetes. Of note, corrections for multiple comparisons adjusting for the total number of statistical tests were not performed in the present study since the analyses were planned before they were conducted.

Our study nonetheless reinforces the potential of MAIT cells as a biomarker of disease progression, now in adults suffering from type 1 diabetes. Our results suggest that MAIT cell alterations in adults with type 1 diabetes are linked with two aspects of the disease, one associated with autoimmunity and another with impaired glucose homeostasis.

## Supplementary information

ESM (PDF 1054 kb)

## Data Availability

Corrplot datasets are available at 10.6084/m9.figshare.c.5328767. All used R scripts are available at https://github.com/MatthieuRouland/Nel-et-al. The datasets generated during and/or analysed during the current study are available from the corresponding author upon reasonable request.

## Abbreviations

BCL-2: B-cell lymphoma 2
DN: Double negative (CD8^−^CD4^−^)
GzB: Granzyme B
LADA: Latent autoimmune diabetes in adults
mAb: Monoclonal antibody
MAIT: Mucosal-associated invariant T
MFI: Mean fluorescence intensity
MR1: MHC-related 1 protein
PBMC: Peripheral blood mononuclear cell
PCA: Principal component analysis
PD1: Programmed cell death 1
PMA: Phorbol myristate acetate

## References

[1] Atkinson MA, Eisenbarth GS, Michels AW (2014). Type 1 diabetes. Lancet.

[2] DiMeglio LA, Evans-Molina C, Oram RA (2018). Type 1 diabetes. Lancet.

[3] Ilonen J, Lempainen J, Veijola R (2019). The heterogeneous pathogenesis of type 1 diabetes mellitus. Nat Rev Endocrinol.

[4] Lehuen A, Diana J, Zaccone P, Cooke A (2010). Immune cell crosstalk in type 1 diabetes. Nat Rev Immunol.

[5] Hull CM, Peakman M, Tree TIM (2017). Regulatory T cell dysfunction in type 1 diabetes: what’s broken and how can we fix it?. Diabetologia.

[6] Bluestone JA, Herold K, Eisenbarth G (2010). Genetics, pathogenesis and clinical interventions in type 1 diabetes. Nature.

[7] Hughes JW, Riddlesworth TD, DiMeglio LA (2016). Autoimmune diseases in children and adults with type 1 diabetes from the T1D Exchange Clinic Registry. J Clin Endocrinol Metab.

[8] Kahaly GJ, Hansen MP (2016). Type 1 diabetes associated autoimmunity. Autoimmun Rev.

[9] Treiner E, Duban L, Bahram S (2003). Selection of evolutionarily conserved mucosal-associated invariant T cells by MR1. Nature.

[10] Ussher JE, Klenerman P, Willberg CB (2014). Mucosal-associated invariant T-cells: new players in anti-bacterial immunity. Front Immunol.

[11] Lantz O, Legoux F (2018). MAIT cells: an historical and evolutionary perspective. Immunol Cell Biol.

[12] Dusseaux M, Martin E, Serriari N (2011). Human MAIT cells are xenobiotic-resistant, tissue-targeted, CD161hi IL-17-secreting T cells. Blood.

[13] Toubal A, Nel I, Lotersztajn S, Lehuen A (2019) Mucosal-associated invariant T cells and disease. Nat Rev Immunol. 10.1038/s41577-019-0191-y 10.1038/s41577-019-0191-y 31308521

[14] Nel I, Bertrand L, Toubal A, Lehuen A (2021) MAIT cells, guardians of skin and mucosa? Mucosal Immunol 1–12. 10.1038/s41385-021-00391-w 10.1038/s41385-021-00391-w PMC7983967 33753874

[15] Corbett AJ, Eckle SBG, Birkinshaw RW (2014). T-cell activation by transitory neo-antigens derived from distinct microbial pathways. Nature.

[16] Ussher JE, Bilton M, Attwod E (2014). CD161++ CD8+ T cells, including the MAIT cell subset, are specifically activated by IL-12+IL-18 in a TCR-independent manner. Eur J Immunol.

[17] Leeansyah E, Svärd J, Dias J (2015). Arming of MAIT cell cytolytic antimicrobial activity is induced by IL-7 and defective in HIV-1 infection. PLoS Pathog.

[18] Loh L, Wang Z, Sant S (2016). Human mucosal-associated invariant T cells contribute to antiviral influenza immunity via IL-18-dependent activation. Proc Natl Acad Sci U S A.

[19] Pavlovic M, Gross C, Chili C, Secher T, Treiner E (2020). MAIT cells display a specific response to type 1 IFN underlying the adjuvant effect of TLR7/8 ligands. Front Immunol.

[20] Rouxel O, Da Silva J, Beaudoin L (2017). Cytotoxic and regulatory roles of mucosal-associated invariant T cells in type 1 diabetes. Nat Immunol.

[21] Le Bourhis L, Martin E, Péguillet I (2010). Antimicrobial activity of mucosal-associated invariant T cells. Nat Immunol.

[22] Rouxel O, Lehuen A (2018). Mucosal-associated invariant T cells in autoimmune and immune-mediated diseases. Immunol Cell Biol.

[23] Gazali AM, Schroderus A-M, Näntö-Salonen K (2020). Mucosal-associated invariant T cell alterations during the development of human type 1 diabetes. Diabetologia.

[24] Rouland M, Beaudoin L, Rouxel O et al (2021) Gut mucosa alterations and loss of segmented filamentous bacteria in type 1 diabetes are associated with inflammation rather than hyperglycaemia. Gut gutjnl-2020-323664. 10.1136/gutjnl-2020-323664 10.1136/gutjnl-2020-323664 33593807

[25] Battaglia M, Atkinson MA (2015). The streetlight effect in type 1 diabetes. Diabetes.

[26] Arif S, Leete P, Nguyen V (2014). Blood and islet phenotypes indicate immunological heterogeneity in type 1 diabetes. Diabetes.

[27] Leete P, Willcox A, Krogvold L (2016). Differential insulitic profiles determine the extent of β-cell destruction and the age at onset of type 1 diabetes. Diabetes.

[28] In’t Veld P (2011). Insulitis in human type 1 diabetes: the quest for an elusive lesion. Islets.

[29] Komulainen J, Kulmala P, Savola K (1999). Clinical, autoimmune, and genetic characteristics of very young children with type 1 diabetes. Diabetes Care.

[30] Karjalainen J, Salmela P, Ilonen J, Surcel HM, Knip M (1989). A comparison of childhood and adult type I diabetes mellitus. N Engl J Med.

[31] Novak J, Dobrovolny J, Novakova L, Kozak T (2014). The decrease in number and change in phenotype of mucosal-associated invariant T cells in the elderly and differences in men and women of reproductive age. Scand J Immunol.

[32] Chen P, Deng W, Li D (2019). Circulating mucosal-associated invariant T cells in a large cohort of healthy Chinese individuals from newborn to elderly. Front Immunol.

[33] Flament H, Rouland M, Beaudoin L (2021). Outcome of SARS-CoV-2 infection is linked to MAIT cell activation and cytotoxicity. Nat Immunol.

[34] R Core Team (2021). R: a language and environment for statistical computing.

[35] Reantragoon R, Corbett AJ, Sakala IG (2013). Antigen-loaded MR1 tetramers define T cell receptor heterogeneity in mucosal-associated invariant T cells. J Exp Med.

[36] Magalhaes I, Pingris K, Poitou C (2015). Mucosal-associated invariant T cell alterations in obese and type 2 diabetic patients. J Clin Invest.

[37] Carolan E, Tobin LM, Mangan BA (2015). Altered distribution and increased IL-17 production by mucosal-associated invariant T cells in adult and childhood obesity. J Immunol.

[38] Touch S, Assmann KE, Aron-Wisnewsky J et al (2018) Mucosal-associated invariant T (MAIT) cells are depleted and prone to apoptosis in cardiometabolic disorders. FASEB J fj201800052RR. 10.1096/fj.201800052RR 10.1096/fj.201800052RR 29957059

[39] Reinert-Hartwall L, Honkanen J, Salo HM (2015). Th1/Th17 plasticity is a marker of advanced β cell autoimmunity and impaired glucose tolerance in humans. J Immunol.

[40] Lee Y, Awasthi A, Yosef N (2012). Induction and molecular signature of pathogenic TH17 cells. Nat Immunol.

[41] Hu D, Notarbartolo S, Croonenborghs T (2017). Transcriptional signature of human pro-inflammatory T H 17 cells identifies reduced IL10 gene expression in multiple sclerosis. Nat Commun.

[42] Provine NM, Amini A, Garner LC (2021). MAIT cell activation augments adenovirus vector vaccine immunogenicity. Science.

[43] Diana J, Simoni Y, Furio L (2013). Crosstalk between neutrophils, B-1a cells and plasmacytoid dendritic cells initiates autoimmune diabetes. Nat Med.

[44] Whitacre CC (2001). Sex differences in autoimmune disease. Nat Immunol.

[45] Beaudoin L, Laloux V, Novak J, Lucas B, Lehuen A (2002). NKT cells inhibit the onset of diabetes by impairing the development of pathogenic T cells specific for pancreatic beta cells. Immunity.

