# Supplementary material for: MAIT cell alterations in adults with recent-onset and long-term type 1 diabetes
Source: Diabetologia. 2021 Aug 4;64(10):2306–21. doi: 10.1007/s00125-021-05527-y (PMC8336671; doi:10.1007/s00125-021-05527-y)
Supplement: Supplementary file 1 — (PDF 1054 kb) [file 125_2021_5527_MOESM1_ESM.pdf]

ESM Table 1: MAIT cell alterations in adults with type 1 diabetes (T1D).

	Men and women		Women	
	**RO T1D**	**LT T1D**	**LT T1D**	**LT T1D and AID**
MAIT cell frequency		$CD8^+$ MAIT cells (-)<br>DN MAIT cells (-)	DN MAIT cells (-)	MAIT cells (-)<br>$CD8^+$ MAIT cells (-)<br>DN MAIT cells (- -)
MAIT cell homeostasis		Ki67 (++)<br>CD127 (+)		CD127 (- -)<br>BCL-2 (- -)
MAIT cell activation	CD25 (++++)<br>IL-4 (++)	CD25 (+++)<br>IL-4 (++)<br>IL-17 (+)	CD25 (+++)	CD69 (+)<br>CD25 (++)
MAIT cell exhaustion		PD1 (++)<br>IL-2 (- - - -)<br>IFN-γ (- -)<br>TNF-α (-)	PD1 (++)<br>IL-2 (- -)<br>IFN-γ (- -)<br>TNF-α (- -)	PD1 (+++)<br>IL-2 (-)<br>IFN-γ (-)<br>TNF-α (- - - -)

The '-' and '+' symbols in brackets respectively represent the decrease or the increase of the frequency of MAIT cells expressing the corresponding parameter compared to healthy individuals.

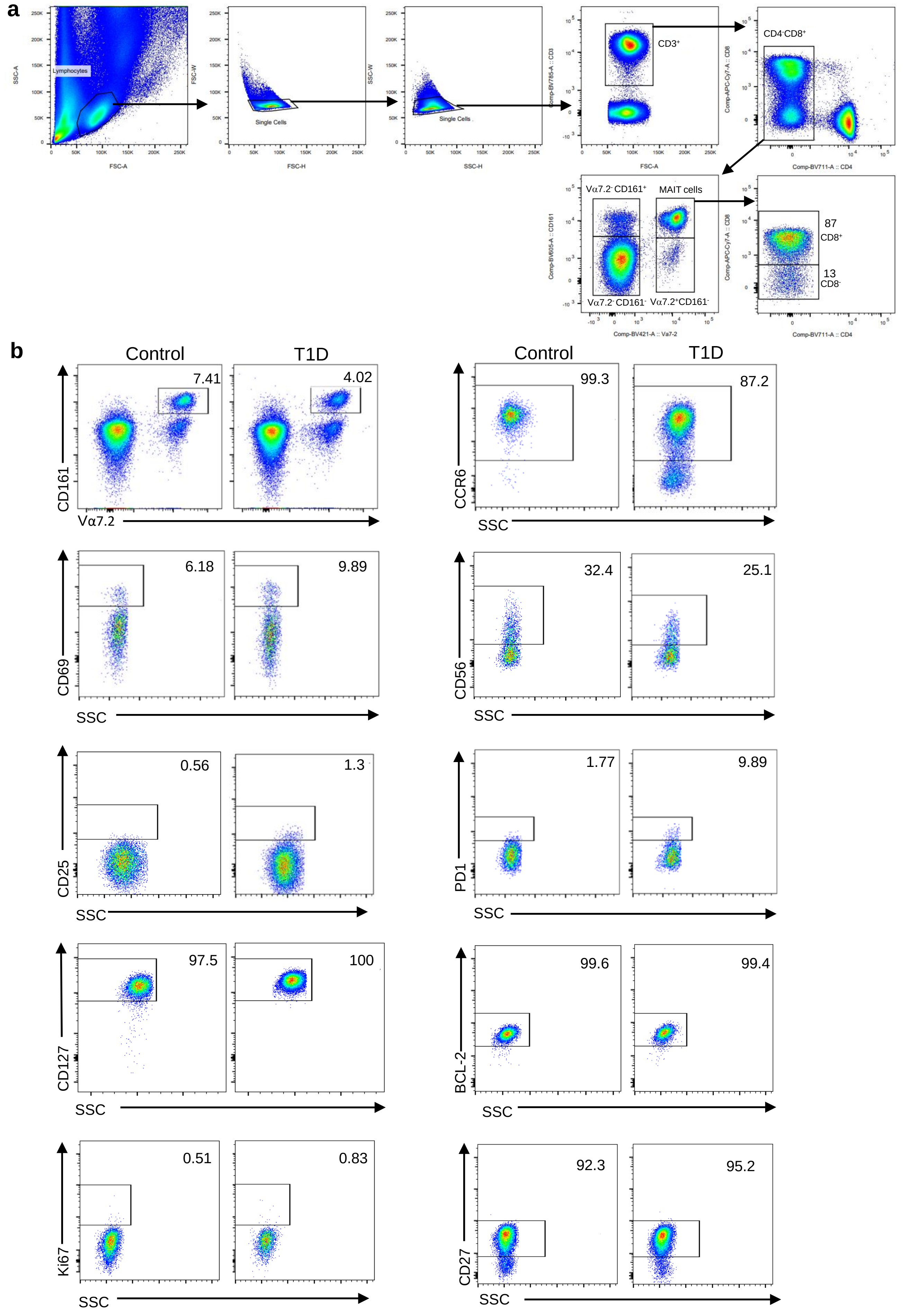

ESM Fig. 1 Flow cytometry gating strategy for MAIT cell identification, activation and exhaustion. Surface gating strategy, in the blood of a healthy adult donor, of circulating MAIT cell frequency used in Fig. 1 and 5 (**a**). Representative dot plot of CCR6^{+}, CD69^{+}, CD56^{+}, CD25^{+}, PD1^{+}, CD127^{+}, BCL-2^{+}, Ki67^{+} and CD27^{+} MAIT cells in blood from a healthy adult donor and an adult patient with long-term type 1 diabetes (**b**). Percentage cell frequencies are shown next to the outlined areas. FSC, Forward scatter. SSC, Side scatter.

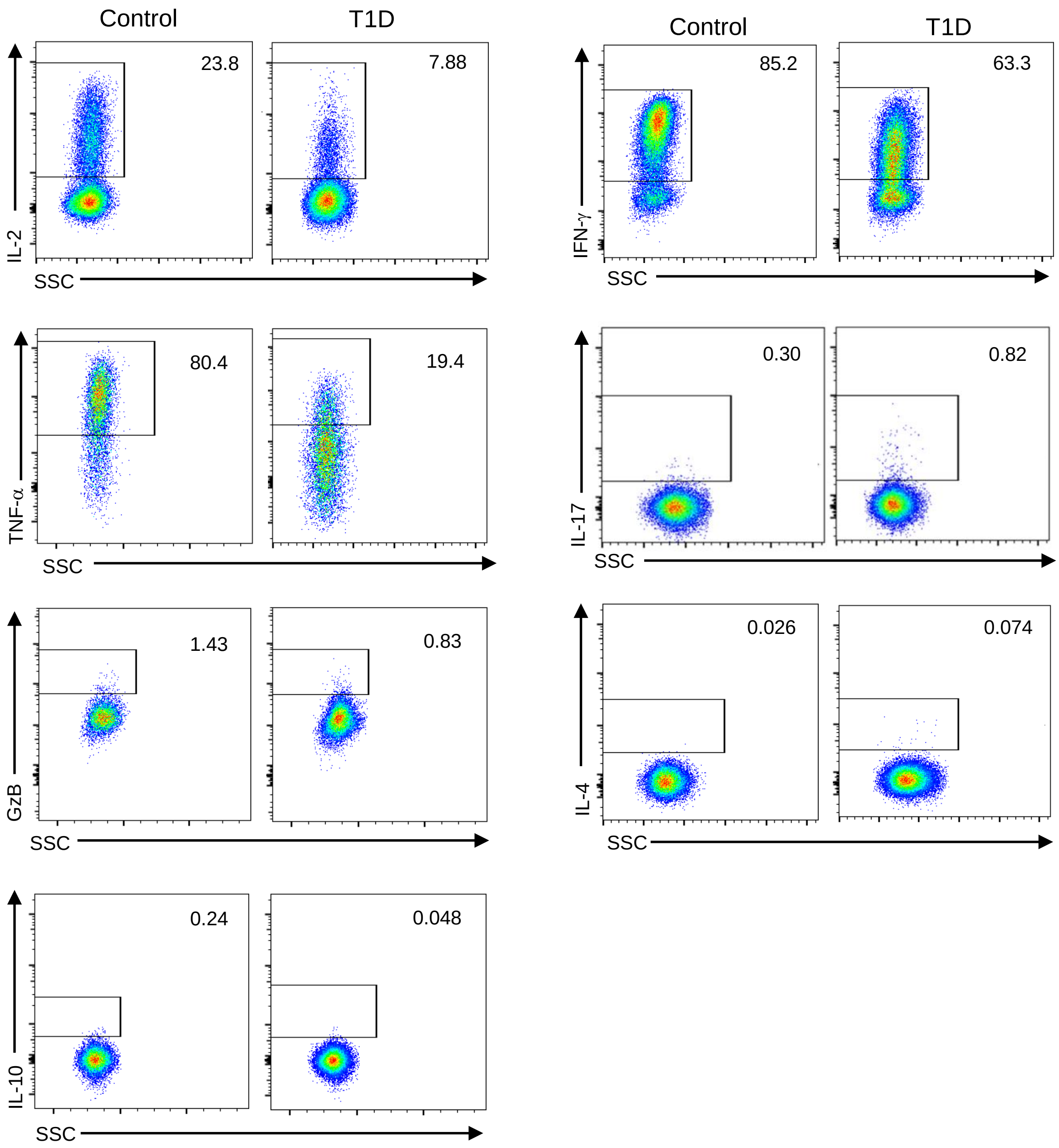

ESM Fig. 2 Flow cytometry gating strategy for MAIT cell function. Representative dot plot of IL-2$^+$, INF-γ$^+$, TNF-α$^+$, IL-17$^+$, GzB$^+$, IL-4$^+$ and IL-10$^+$ MAIT cell in blood from a healthy adult donor and an adult patient with long-term type 1 diabetes. Percentage cell frequencies are shown next to the outlined areas. SSC, Side scatter.

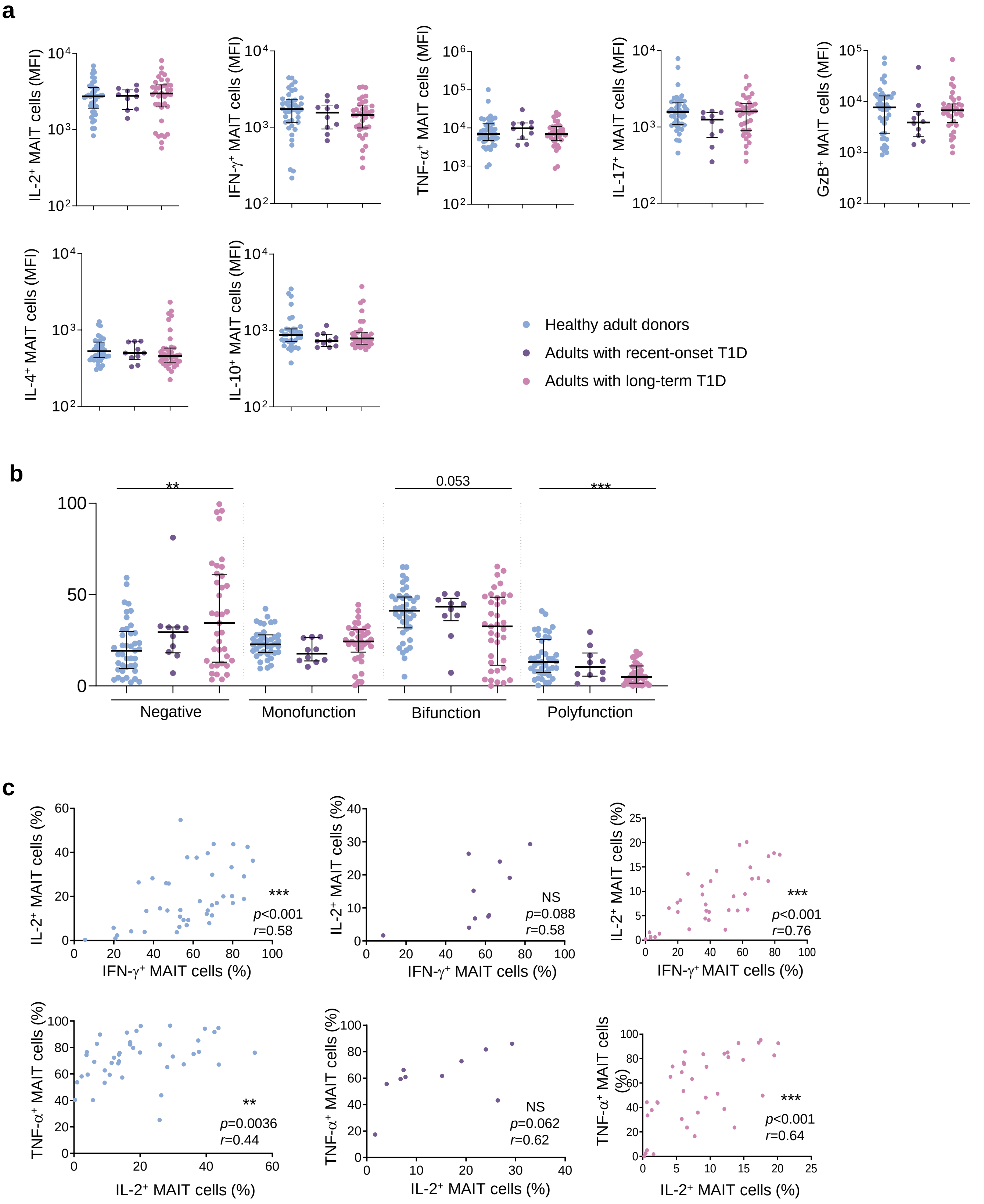

ESM Fig. 3 Functional analysis of MAIT cells in adults with recent onset and long-term type 1 diabetes. Flow cytometry analysis of IL-2⁺, IFN-γ⁺, TNF-α⁺, IL-17⁺, Granzyme B⁺ (GzB), IL-4⁺ or IL-10⁺ MAIT cell Mean Fluorescence Intensity (MFI) among total MAIT cells in healthy adult donors (n=42), adult patients with recent onset (RO) (n=10) or long-term (LT) (n=37) type 1 diabetes (**a**). Frequency of cumulative Th1 cytokines, IL-17, and GzB from Fig. 2a with negative as 0, monofunctional as 1, bifunctional as 2 and polyfunctional from 3 up to 5 different cytokine production by MAIT cells in healthy donors (n=42), patients with RO (n=10) or LT (n=37) type 1 diabetes (**b**). Correlations between IL-2⁺ MAIT cell and IFN-γ⁺ MAIT cell frequencies, between TNF-α⁺ MAIT cell and IL-2⁺ MAIT cell frequencies in healthy donors (n=42), patients with RO (n=10) or LT (n=37) type 1 diabetes (**c**). Each symbol represents an individual subject. Small horizontal lines indicate median with interquartile range. * *p* <0.05, ** *p* <0.01, *** *p* <0.001 (non-parametric two-tailed Mann-Whitney test or Spearman's correlation test).

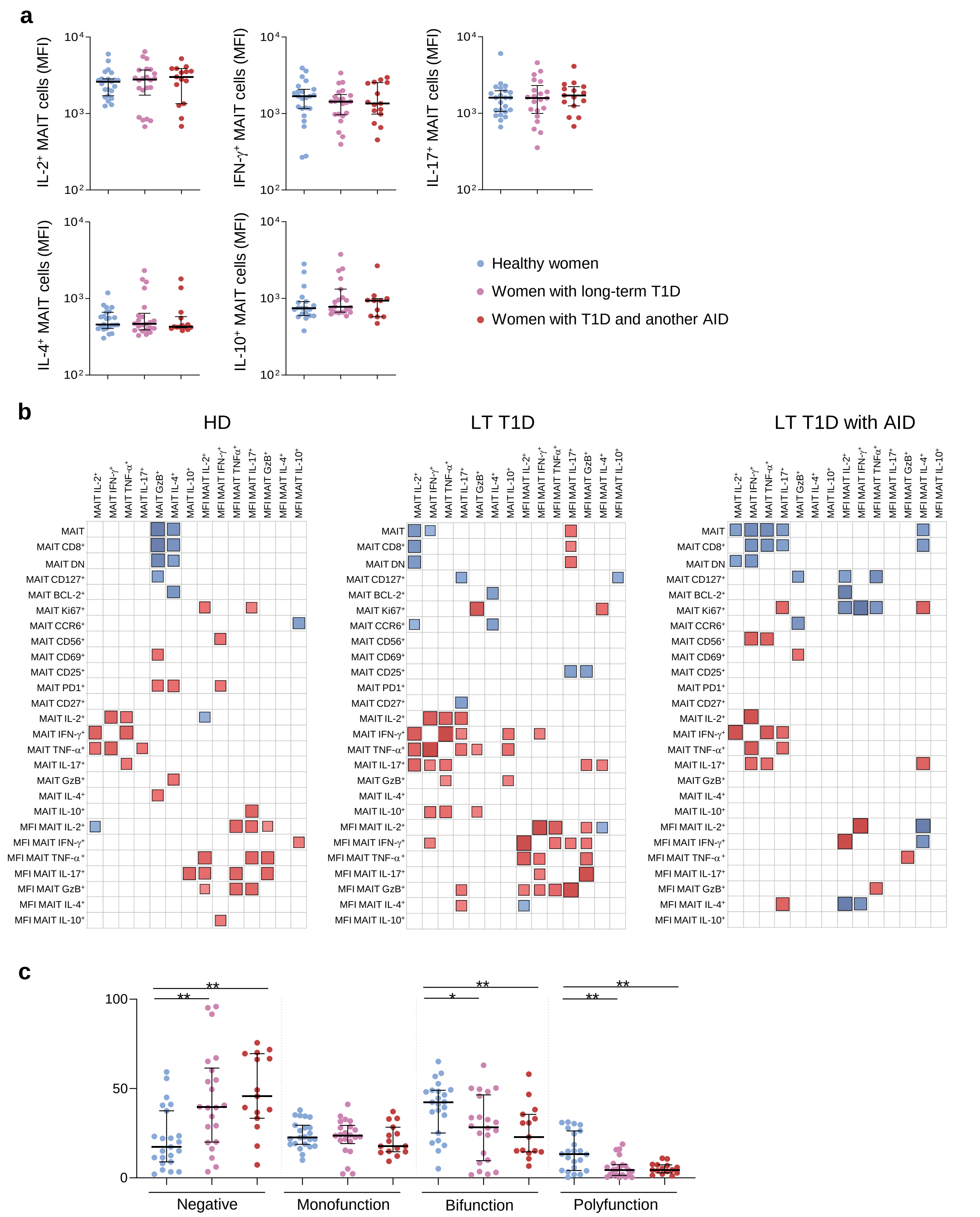

ESM Fig. 4 Functional analysis of MAIT cells in women with long-term type 1 diabetes and long-term type 1 diabetes with another autoimmune disease. Flow cytometry analysis of IL-2+, IFN-γ+, IL-17+, IL-4+ or IL-10+ MAIT cell Mean Fluorescence Intensity (MFI) among total MAIT cells in healthy women donors (n=22), women with long-term (LT) (n=23) type 1 diabetes or LT type 1 diabetes with another auto-immune disease (AID) (n=15) (**a**). Correlogram of circulating MAIT cell frequency, phenotype, function and MFI presented in Fig. 5a, 6a and in ESM Fig. 4a in healthy women donors (n=15-30), women patients with LT type 1 diabetes (n=14-27) or LT type 1 diabetes with another AID (n=9-17). Only significant Spearman's correlation coefficients are represented by color intensity and square size (**b**). Frequency of cumulative Th1 cytokines, IL-17, and GzB production from Figure 6a with negative as 0, monofunctional as 1, bifunctional as 2 and polyfunctional from 3 up to 5 different cytokine expression on MAIT cells in healthy women donors (n=22), women with LT type 1 diabetes (n=23) or LT type 1 diabetes with another AID (n=15) (**c**). * *p* <0.05, ** *p* <0.01 (non-parametric two-tailed Mann-Whitney tests or Spearman's correlation test).
